# Live tissue microbiota and bacterial translocation: mechanisms and translational perspectives in cardiometabolic diseases

**DOI:** 10.1007/s11154-026-10017-w

**Published:** 2026-02-27

**Authors:** Matthieu Minty, Sylvie Lê, Myriam Addi, Thibault Canceill, Charlotte Thomas, Pascale Loubieres, Rémy Burcelin, Vincent Blasco-Baque

**Affiliations:** 1 Health Faculty of Toulouse, Toulouse University, 3 Chemin des Maraîchers, Toulouse, 31062, Cedex 9 France; 2 Odontology Department, Toulouse Universitary Hospital, 2 Rue de Viguerie, Toulouse, 31100 France; 3 InCOMM (Intestine Clinical Oralomics Microbiota & Metabolism) UMR1297 Inserm, Université de Toulouse, Institute of Metabolic and Cardiovascular Diseases (i2MC), 1 Avenue Jean Poulhes, Toulouse, 31432 France

**Keywords:** Bacterial translocation, Tissue microbiota, Cardiometabolic diseases, Host-microbiome interaction, Precision medicine

## Abstract

The long-standing view of sterile internal tissues has been challenged by accumulating evidence that microbial material, and under specific conditions, viable bacteria may translocate from the gut and oral cavity into metabolic and cardiovascular tissues. This review synthesizes current knowledge on the mechanisms underlying bacterial translocation and its implications for cardiometabolic disease, complemented by original experimental data. Dysbiosis and epithelial barrier disruption facilitate the passage of microbial components and, in some settings, culturable bacteria across mucosal surfaces, triggering local and systemic inflammation. In our murine models, high-fat feeding markedly increased the recovery of culturable bacteria from visceral adipose tissue and spleen, with tissue-specific bacterial signatures enriched in pro-inflammatory taxa. GFP-labelled *E. coli* translocated more abundantly under metabolic stress, while CD14 deficiency significantly reduced dissemination, highlighting the role of LPS–CD14 signaling. Clinical studies consistently report bacterial DNA and, in some cases, viable bacteria in adipose tissue, liver, atherosclerotic plaques, and heart valves, correlating with immune cell infiltration, cytokine production, and disease severity. However, because internal organs are low-biomass environments, interpretation requires stringent contamination controls and orthogonal evidence of viability and localization. Together, these findings support bacterial translocation as a plausible contributor to chronic low-grade inflammation, insulin resistance, and cardiometabolic pathology. Targeting microbial translocation through barrier reinforcement, microbiota modulation, and metabolite inhibition may offer novel preventive and therapeutic strategies that warrant careful validation.

## Introduction

Cardiometabolic and cardiovascular diseases have emerged as leading public health challenges over the past decades, driven by the increasing prevalence of obesity, type 2 diabetes, and hypertension, despite advances in the management of conventional risk factors [1]. This epidemiological trend strongly suggests the contribution of additional, non-traditional mechanisms underlying disease initiation and progression. Among these, disturbances in the human microbiota, a complex and dynamic ecosystem inhabiting notably the gut and oral cavity, have attracted increasing scientific attention [2, 3]. A growing body of evidence supports the notion that microbial dysbiosis, defined as alterations in the diversity and composition of commensal communities, can trigger systemic low-grade inflammation, immune activation, and metabolic dysfunction [4, 5]. One of the most compelling hypothesis emerging from this field is bacterial translocation, defined as the passage of live bacteria or their molecular components across mucosal barriers into otherwise sterile tissues and organs [6]. Initially described as a rare phenomenon restricted to critical illness or sepsis, bacterial translocation is now recognized as a more frequent and physiologically relevant process that can be amplified under conditions of dysbiosis and barrier disruption in many systemic diseases [7, 8].

While the primary focus of translocation studies has traditionally been intestinal permeability, recent findings highlight the oral mucosa as an additional and underappreciated source of microbial dissemination, particularly in the context of periodontal disease [9]. The overrepresentation of Gram-negative anaerobes in both gut and oral dysbiosis leads to increased production of lipopolysaccharide (LPS), a potent endotoxin that binds Toll-like receptor 4 (TLR4) and CD14, activating downstream inflammatory cascades involving MyD88 and NF-κB [10–12]. These pathways promote the secretion of pro-inflammatory cytokines such as TNF-α, IL-6, and IL-1β, which in turn impair the structural integrity of epithelial tight junctions and exacerbate barrier dysfunction [13, 14]. In parallel, oral bacteria such as Porphyromonas gingivalis, Gram-negative bacteria, have been shown to invade periodontal tissues by producing proteolytic enzymes that degrade adhesion molecules and facilitate microbial infiltration into systemic circulation [15]. Notably, this mechanism has been implicated in the pathogenesis of atherosclerosis, insulin resistance, and type 2 diabetes. Together, these insights indicate that both intestinal and oral dysbiosis are associated with bacterial translocation and systemic inflammation, thereby establishing a mechanistic link between mucosal microbial ecosystems and distant metabolic and cardiovascular tissues [16–18].

Beyond the well-described concept of metabolic endotoxemia, which refers to the translocation of bacterial components (metafactors) such as LPS, an original hypothesis proposes that whole, viable bacteria can cross mucosal barriers and persist in internal tissues, forming metabolically active microbial communities—referred to as live tissue microbiota [19]. Pioneering studies by Ahne et al. [20], Amar & Koren et al. [21], and more recently Minty et al. [22], have demonstrated the presence of bacterial DNA within adipose tissue and liver in human cohorts. This concept challenges the long-standing paradigm of sterile internal organs and suggests that bacterial colonization of metabolic tissues could act as a chronic trigger for immune activation and metabolic disruption. Although many aspects of this phenomenon remain poorly understood, the integration of animal experiments and clinical observations supports the notion that bacterial translocation from oral and intestinal microbiota and tissue colonization may represent novel determinants of cardiometabolic disease risk. Importantly, dietary and environmental factors—including vitamin D deficiency [23], chronic alcohol consumption [24], and gluten intake [25]—have been shown to modulate barrier function and microbial ecology, thus influencing the propensity for bacterial dissemination.

Accordingly, the objectives of this review are: (1) to synthesize current evidence on the mechanisms governing bacterial translocation; (2) to examine the emerging concept of live tissue microbiota; (3) to integrate original experimental data from murine models of metabolic disease realised in our laboratory; and (4) to discuss the clinical implications of microbial translocation for the prevention and treatment of cardiometabolic disorders. Additionally (5), we will explore how environmental and dietary modulators impact mucosal integrity and systemic inflammation, potentially offering new therapeutic avenues.

## Mechanisms and pathophysiology of bacterial translocation

The human gastrointestinal and oral mucosa harbour dense and functionally diverse microbial communities that play essential roles in metabolic regulation, epithelial homeostasis, and immune tolerance [26]. Under physiological conditions, this ecosystem is confined to the luminal compartment by a multilayered defence system comprising the mucus layer, secretory immunoglobulin A (sIgA), antimicrobial peptides, and a network of tight junction proteins that maintain epithelial integrity [27].

Disruption of this balanced state—dysbiosis—can result from various factors, including Western-type diets rich in saturated fats and refined carbohydrates [28], micronutrient deficiencies, chronic alcohol consumption, infections, and antibiotic exposure [29]. Dysbiosis is typically characterized by a loss of microbial diversity, depletion of beneficial commensals such as *Akkermansia muciniphila*, *Bifidobacterium*, and *Lactobacillus*, and an overrepresentation of Gram-negative anaerobes such as *Prevotella*, *Fusobacterium*, and *Clostridium* [30, 31].

This compositional shift alters microbial metabolism, notably reducing the production of short-chain fatty acids (SCFAs) like butyrate, which are critical for nourishing colonocytes and sustaining tight junction expression [32]. As highlighted by Parada Venegas and colleagues, decreased SCFA availability not only deprives epithelial cells of energy but also impairs the activation of G-protein-coupled receptors such as GPR41, GPR43, and GPR109A, which are essential for regulating inflammation and maintaining barrier integrity [32]. The ensuing impairment of barrier integrity increases mucosal permeability, facilitating the passage of bacteria and microbial components into underlying tissues—a process referred to as bacterial translocation [14]. As demonstrated by Wells and Erlandsen [33], experimental models show that disruption of epithelial tight junctions and mucosal injury markedly promote the dissemination of viable bacteria and endotoxins into mesenteric lymph nodes and distant organs. Although most mechanistic insights arise from the gastrointestinal tract, analogous processes occur at other epithelial barriers. In the lung, metatranscriptomic studies in severe pneumonia demonstrate concordant detection of bacterial, viral, and fungal taxa in bronchoalveolar lavage fluid and blood, indicating pulmonary-to-systemic translocation during barrier breach [34]. Likewise, cutaneous barrier disruption in psoriasis facilitates absorption of microbial products such as LPS, contributing to systemic inflammatory signatures [35]. Consistent with these findings, recent studies have further confirmed that compromised barrier function allows microbial products to enter systemic circulation and contribute to inflammation and disease progression [14]. At the molecular level, lipopolysaccharide (LPS), the major component of Gram-negative bacterial outer membranes, acts as a pivotal mediator of this phenomenon [36, 37]. LPS binds to CD14–TLR4 complexes expressed on immune and epithelial cells, triggering downstream signalling cascades via MyD88 and TIRAP adaptors [38]. These pathways converge on NF-κB (transcriptional factor) activation, resulting in the up-production of pro-inflammatory cytokines including TNF-α, IL-6, IL-1β, and IFNγ [39]. These cytokines not only sustain local immune activation but also impair epithelial regenerative capacity and disrupt intercellular cohesion [40]. This engagement activates two main pathways: a MyD88-dependent route leading to NF-κB and MAPK activation and rapid production of proinflammatory cytokines, and a TRIF-dependent pathway promoting IRF3 activation and type I interferon expression [36, 38]. These coordinated signalling cascades are essential for initiating innate immune responses against Gram-negative bacteria. However, excessive or uncontrolled activation can contribute to chronic inflammation and tissue injury [14]. In addition to LPS, *Porphyromonas gingivalis* expresses other potent virulence factors such as fimbriae and gingipains that act as key metafactors in epithelial disruption and immune activation. Fimbriae (notably FimA) engage TLR2 and integrins (e.g., α5β1), initiating NF-κB and MAPK signalling pathways, which drive IL-8 and TNF-α secretion and promote leukocyte recruitment and inflammation [41, 42]. Fimbriae also facilitate bacterial adhesion and invasion by binding to extracellular matrix proteins like fibronectin and interacting with epithelial cadherins, destabilizing intercellular junctions. Gingipains (RgpA, RgpB, and Kgp), cysteine proteases secreted by *P. gingivalis*, degrade junctional proteins such as JAM-1, occludin, and ZO-1, as well as basement membrane components (laminin, collagen IV), resulting in increased paracellular permeability [15, 43]. Gingipains also cleave immune receptors (CD14, complement C5) and modulate host cell signalling by degrading PI3K/Akt and β-catenin pathways, amplifying epithelial damage and inflammatory [44].

The structural basis of epithelial barrier integrity relies on tight junction (TJ) complexes, composed of transmembrane proteins (claudins, occludins, and junctional adhesion molecules [JAMs]) and cytoplasmic scaffolding proteins such as ZO-1, ZO-2, and ZO-3, which anchor TJs to the actin cytoskeleton [45]. González-Mariscal et al. have emphasized that tight junctions (TJs) are not static seals but highly dynamic multiprotein complexes whose composition, nanoscale organization and barrier properties are continuously remodelled to adapt to physiological and inflammatory cues [46]. Their work underlines that TJ function depends on coordinated signalling networks that control the transcriptional regulation of TJ components, their trafficking/endocytosis and recycling, and their anchoring to the perijunctional actomyosin ring [46].

Inflammatory mediators—including TNF-α, IL-6, and IL-1β—not only directly downregulate TJ protein expression but also activate intracellular signalling pathways such as myosin light chain kinase (MLCK), Rho-associated kinase (ROCK), and NF-κB [47]. These pathways induce phosphorylation of myosin light chains and reorganization of the perijunctional actin cytoskeleton, leading to cytoplasmic redistribution and internalization of tight junction components like claudins and occludin. The resulting structural disassembly creates a “leaky” epithelium that permits paracellular translocation of microbial antigens and viable bacteria [47].

Mechanistically, TNF-α and IL-1β trigger NF-κB–dependent MLCK induction, resulting in pMLC and increased actomyosin contractility at the apical junctional complex. In line with this mechanism, He et al. [48] showed that TNF-α induces epithelial barrier “leaks” through NF-κB–dependent MLCK induction, coupled to cytoskeletal remodelling and redistribution of TJ proteins, thereby driving a marked increase in paracellular permeability [48]. This mechanical tension promotes junctional opening and drives the redistribution and internalization of transmembrane TJ proteins (notably occludin and specific claudins), often via clathrin- or caveolin-dependent endocytic routes, with concomitant disengagement of scaffold proteins such as ZO-1. In parallel, pro-inflammatory signalling activates the RhoA/ROCK pathway, reinforcing stress-fibre formation and F-actin remodelling, which further destabilizes TJ strands and amplifies paracellular leak. Consistently, Huang et al. [49] demonstrated in an intestinal epithelial model that activation of RhoA/ROCK signalling induces F-actin reorganization, downregulates key TJ proteins (occludin, claudin-1/3, ZO-1), decreases TEER and increases FITC-dextran flux, providing direct evidence that RhoA/ROCK-driven cytoskeletal tension is sufficient to trigger barrier disruption [49]. Importantly, inflammatory cytokines can also qualitatively reprogram TJ composition: Suzuki et al. [50] demonstrated that IL-6 increases epithelial permeability by inducing the pore-forming claudin-2, through MEK/ERK and PI3K signalling and a Cdx2-dependent transcriptional programme, establishing claudin-2 switching as a direct molecular driver of cytokine-induced “leaky” junctions [50]. Together, these convergent events result in a functional “loosening” and qualitative reshaping of TJs, thereby facilitating the paracellular passage of microbial products—and, under severe barrier disruption, viable bacteria, into underlying tissues and the circulation.

Beyond these structural alterations, the assembly and disassembly of tight junctions are dynamically regulated by a complex network of intracellular signalling pathways that integrate environmental, metabolic, and inflammatory cues [45]. Under physiological conditions, these mechanisms maintain selective permeability essential for nutrient absorption while preventing pathogen translocation [51]. Inflammatory stress disrupts this equilibrium. Protein kinase C, myosin light chain kinase (MLCK), Rho GTPases, and mitogen-activated protein kinases cooperate to orchestrate cytoskeletal remodelling and the turnover of TJ proteins [52–54]. For example, TNF-α and IL-1β induce MLCK transcription via NF-κB activation, leading to phosphorylation of myosin light chains and contraction of the perijunctional actin-myosin ring, which mechanically widens intercellular spaces [50, 55]. Simultaneously, RhoA and ROCK pathways modulate actin polymerization dynamics and contribute to tight junction disassembly [56, 57].

In addition to promoting cytoskeletal contraction, NF-κB activation critically impairs epithelial barrier function by downregulating key tight junction proteins. Ma et al. [58] showed that TNF-α exposure in Caco-2 monolayers significantly reduces both mRNA and protein levels of occludin and ZO-1, as demonstrated by RT-PCR and immunoblot analyses. Immunofluorescence confirmed that this loss is accompanied by disrupted junctional localization, with occludin and ZO-1 displaying a discontinuous pattern along cell borders. Importantly, pharmacological inhibition of NF-κB prevented these changes, preserving tight junction integrity and paracellular barrier function [58]. These results indicate that NF-κB activation acts through a dual mechanism: inducing MLCK-dependent contraction and simultaneously repressing transcription of tight junction components, together leading to compromised epithelial permeability [58]. These findings are further supported by more recent studies demonstrating that modulation of NF-κB activity directly influences tight junction protein expression. For example, activation of the NF-κB pathway by PIK3R3 overexpression has been shown to suppress ZO-1 expression in epithelial cells [47], whereas inhibition of NF-κB signalling preserves or even restores tight junction integrity. In experimental models of intestinal inflammation, compounds such as copper–luteolin complexes [59] and phytochemicals [60, 61] effectively inhibit NF-κB activation, resulting in increased levels of occludin, claudin-1, and ZO-1 and improved barrier function. Collectively, these observations highlight the fact that targeting NF-κB not only attenuates inflammatory signalling but also stabilizes the transcriptional and structural components of the epithelial barrier. Experimental models have demonstrated that pharmacologic inhibition of MLCK or Rho kinase effectively restores tight junction integrity and limits cytokine-induced increases in paracellular permeability. Specifically, Wang et al. [62] showed that combined treatment with TNF-α and interferon-γ synergistically upregulates MLCK expression and increases myosin light chain phosphorylation in intestinal epithelial monolayers, leading to marked reductions in transepithelial electrical resistance and enhanced paracellular flux [62]. Importantly, pharmacologic blockade of MLCK or Rho-associated kinase preserved tight junction structure and function, highlighting the critical role of actomyosin contraction in cytokine-driven barrier disruption [62]. Consistent with these findings, Clayburgh et al. [63] demonstrated in vivo that T cell activation induces MLCK-dependent barrier dysfunction, which can be reversed by selective MLCK inhibition [63].

In addition to these paracellular mechanisms, specialized epithelial and immune cells also participate in microbial sampling and translocation. For example, microfold (M) cells within Peyer’s patches, as well as CX3CR1⁺ macrophages and CD103⁺ dendritic cells, can actively transport bacteria or their antigens across the intestinal barrier to mesenteric lymph nodes—a process essential for mucosal immune surveillance but potentially subverted under conditions of dysbiosis [64, 65]. Importantly, however, bacterial translocation is not invariably associated with overt systemic infection. In some cases, viable microorganisms can persist in a dormant or low-replication state within internal compartments, including the liver, adipose depots, pancreas, and cardiovascular tissues [21, 66, 67].

This concept of a *live tissue microbiota* broadens the classical paradigm of endotoxemia by suggesting that metabolically active or quiescent bacteria may sustain chronic low-grade inflammation and directly contribute to disease progression [21]. This emerging model is substantiated by converging experimental and clinical evidence. In animal studies, Cani and colleagues [37] demonstrated that high-fat diet feeding markedly increases intestinal permeability, leading to elevated circulating levels of lipopolysaccharide—a phenomenon referred to as metabolic endotoxemia—which in turn promotes macrophage infiltration and inflammatory gene expression in adipose tissue. Extending these observations, Amar et al. [21] provided direct evidence of viable commensal bacteria translocating across the gut barrier and accumulating within visceral adipose depots and the liver, where they interact with innate immune cells and drive local cytokine production. Further mechanistic insights were offered by Boutens and Stienstra [68], who reviewed how such bacterial components and metabolites contribute to the polarization of adipose tissue macrophages toward pro-inflammatory M1 phenotypes characterized by increased expression of TNF-α, IL-6, and CD11c. Complementary findings in humans have reinforced the translational relevance of these processes. Massier et al. [66] detected bacterial DNA signatures in adipose tissue biopsies from obese individuals and patients with type 2 diabetes and were able to culture viable bacteria from these depots, supporting the notion of persistent tissue colonization. In parallel, studies by Koren et al. [69] and Lehtiniemi et al. [70] identified bacterial DNA derived from oral and gut taxa within atherosclerotic plaques, suggesting that translocated microbes may also contribute to vascular inflammation and plaque progression and its rupture. Building on these findings, Minty et al. [22] provided further evidence that depot-specific bacterial signatures in adipose tissue are associated not only with metabolic status, but also with longitudinal weight loss outcomes following bariatric surgery. Their study identified predictive microbial DNA patterns within visceral and subcutaneous fat that correlated with postoperative weight trajectories, highlighting a functional link between tissue microbiota composition and metabolic response [22].

Beyond the gut and oral cavity, similar mechanisms of barrier failure and microbe–host interaction operate at other epithelial surfaces. In the lung, inflammatory injury and disruption of tight junctions during severe pneumonia permit microbial and endotoxin spillover from the airways into the bloodstream [34, 71, 72], while in the female reproductive tract, mucosal barrier weakening and dysbiosis in gynecologic disorders are increasingly linked to systemic immune perturbation even in the absence of overt bacteremia [73]. Likewise, cutaneous barrier defects in inflammatory skin diseases facilitate systemic exposure to microbial ligands such as LPS and are associated with low-grade systemic inflammation and extra-cutaneous comorbidities [35, 74]. Together, these observations support the view that gut, oral, pulmonary, reproductive, and cutaneous epithelia act as interconnected microbial reservoirs that can contribute either via episodic translocation of viable microbes or through the chronic release of microbial products and metabolite alterations that propagate systemic inflammatory signalling.

Collectively, these findings delineate a mechanistic continuum in which dysbiosis-driven barrier dysfunction facilitates bacterial translocation, persistent colonization of metabolic tissues, and sustained systemic inflammation. This interplay likely constitutes a pivotal pathophysiological link between microbiota perturbations and the development of cardiometabolic diseases.

## The concept of live tissue microbiota: from translocation to colonization

Traditionally, internal tissues and organs—including adipose depots, liver, pancreas, and cardiovascular structures—were regarded as sterile under physiological conditions. This paradigm originated primarily from the limited sensitivity of culture-based methods, along with the long-standing assumption that any microbial signal detected in tissue samples merely reflected procedural contamination or transient bacteremia [75]. However, advances in high-throughput sequencing, rigorous contamination controls, and culture-enrichment techniques have challenged this view, revealing the consistent detection of bacterial DNA, RNA transcripts, and, in some studies, viable bacteria within metabolic and cardiovascular tissues [66, 69, 76].

The emerging concept of a *live tissue microbiota* proposes that certain bacteria can not only translocate across disrupted mucosal barriers but also persist and establish metabolically active or quiescent communities within host tissues [66, 77]. Unlike the classical model of metabolic endotoxemia—primarily driven by the systemic diffusion of soluble microbial products such as lipopolysaccharide (LPS)—this hypothesis suggests that whole bacteria act as chronic reservoirs of immunostimulatory signals, engaging in sustained crosstalk with resident immune and parenchymal cells [66].

Pioneering studies have provided compelling evidence supporting this paradigm shift. For example, Koren et al. [69] performed deep sequencing of human atherosclerotic plaques and demonstrated enrichment of bacterial DNA corresponding predominantly to *Proteobacteria* and *Bacteroidetes* taxa, suggesting translocation from gut and oral niches. In a landmark study, Ha et al. [77] combined bacterial culture, 16 S rRNA gene sequencing, and immunofluorescence to show that viable *Enterobacteriaceae* and *Proteobacteria* colonize creeping mesenteric fat in Crohn’s disease patients, directly linking bacterial dissemination to adipose tissue expansion. In murine models of obesity, Amar et al. [21] and Anhê et al. [76] elegantly demonstrated that orally administered fluorescently labelled commensal bacteria could be recovered alive from visceral adipose tissue and liver, confirming that translocation results in genuine tissue colonization rather than passive DNA leakage. Notably, Anhê et al. also observed that antibiotic pretreatment significantly reduced bacterial load in these tissues, further supporting an active dissemination process rather than contamination [76].

Several mechanistic pathways likely underpin the persistence and adaptation of bacteria within internal tissues. We draw here on the landmark syntheses by Belkaid and Hand [26] and by Macpherson and Harris [78], which provide a unifying conceptual framework for persistence mechanisms following microbial translocation. Within this framework, one major route of persistence is the formation of structured biofilm-like microcolonies. In such aggregates, bacteria are embedded in an extracellular matrix (polysaccharides, proteins and eDNA) that limits opsonization and physical access of phagocytes, attenuates antimicrobial peptide penetration, and creates local metabolic gradients that favour slow-growing, stress-tolerant subpopulations. This mechanism is supported by original in vivo work showing that biofilm microcolonies can actively prevent macrophage phagocytosis and promote chronic survival within tissues [79]. A second route involves intracellular persistence. As emphasized by Macpherson and Harris [78], translocated bacteria may be internalized by professional or non-professional phagocytes and adopt low-replicative “persister/SCV-like” states, enabling resistance to phagolysosomal killing and reduced antibiotic susceptibility. Primary studies have demonstrated such phenotype switching toward small-colony variants that are specifically adapted for intracellular survival and associated with chronic infection relapse [80]. Moreover, intracellular persistence is not restricted to immune cells: endothelial cells can constitute long-lived intracellular niches, where bacteria survive for days to weeks in slowly replicating or non-replicative states, consistent with immune-shielded reservoirs capable of reseeding dissemination [81]. To conclude, these complementary mechanisms, biofilm-like protected aggregates and intracellular persistence across immune and non-immune compartments, provide a coherent and biologically plausible basis for transient or longer-term bacterial survival within internal tissues.

We suppose that once established, tissue-resident bacteria engage pattern recognition receptors—including TLR2, NOD1, and NOD2—on innate immune cells, inducing sustained low-grade inflammatory cytokine production [82, 83]. Importantly, compositional analyses consistently demonstrate that the tissue-associated microbiota exhibits distinct taxonomic profiles compared to luminal or adjacent communities, arguing against indiscriminate contamination [84, 85]. For example, Massier et al. analyzed adipose tissue samples from obese patients and detected a predominance of *Streptococcaceae* and *Enterobacteriaceae*, which differed significantly from fecal microbiota profiles [66]. Similarly, Ha et al. demonstrated that mesenteric adipose tissue harbored bacterial taxa absent or present only in very low abundance in matched stool samples, suggesting selective translocation and niche-specific colonization [77].

Next-generation sequencing technologies have significantly increased the sensitivity of microbiome research, enabling the detection of microbial DNA in low-biomass tissue samples. However, this enhanced sensitivity also increases the risk of detecting contaminant DNA or non-viable microbes, potentially compromising the biological interpretation of results. To address this, many researchers emphasize the importance of complementing DNA-based detection with indicators of microbial viability or activity, such as ribosomal RNA expression, culture-based recovery, or in situ imaging techniques. For example, Eisenhofer et al. [75] provided a comprehensive framework to minimize contamination in low-biomass microbiome studies, recommending the use of negative controls, quantitative assessments of microbial load, and stringent decontamination procedures to help validate biological relevance. As Stinson et al. [86] emphasize, rigorous decontamination protocols and complementary evidence of microbial activity—such as RNA expression, culture recovery, or imaging—are crucial for determining biological relevance [86]. Among such complementary methods, fluorescence in situ hybridization (FISH) combined with spectral or confocal microscopy has been particularly valuable. Valm et al. [87] demonstrated that advanced FISH-based imaging enables the spatial localization of bacterial communities within tissue structures, offering critical insights into microbial organization and host–microbe interactions [87].

Over the past decade, methodological advances have substantially improved the reliability of microbial detection in low biomass tissues. Improvements in surgical asepsis now include the use of physically separated sampling and microbiology workspaces, sterile single use instruments for each specimen, and the systematic incorporation of intra operative blanks and reagent only controls, which together reduce the likelihood of environmental or procedural contamination. In parallel, culture-based approaches have undergone a profound transformation, with extended anaerobic and low oxygen incubation, the use of enriched or host mimicking media, and the systematic variation of culture conditions, all contributing to an expanded recovery of slow growing or previously uncultivable taxa. Pioneering culturomics studies have shown that increasing the diversity of culture conditions can dramatically increase the number and diversity of viable bacteria isolated from human samples, thereby reshaping our view of the human microbiome [88]. Complementary work by Browne et al. has further demonstrated that rationally designed media and targeted cultivation strategies can recover numerous previously uncultured gut bacterial species, highlighting how methodological refinement directly translates into an expanded catalogue of viable human associated microbes [89]. These technological developments collectively redefine what can be detected and provide a more rigorous framework for evaluating whether viable microorganisms may be present in internal organs.

Beyond technical considerations, the biological implications of a live tissue microbiota are substantial. Persistent bacterial signals in metabolic tissues may act as a continuous source of pathogen-associated molecular patterns (PAMPs), sustaining chronic low-grade inflammation and contributing to adipocyte dysfunction, insulin resistance, and vascular injury. For example, Koh et al. [90] demonstrated that microbial metabolites like imidazole propionate impair insulin signalling by activating the mTORC1 pathway in hepatocytes, linking microbial activity to systemic metabolic dysregulation [90]. Additionally, microbial components have been detected in atherosclerotic plaques and arterial tissues, suggesting that bacterial persistence may influence plaque stability and local immune responses. As reviewed by Witkowski et al. [91], gut microbiota dysbiosis and the translocation of bacterial products into circulation may drive endothelial dysfunction and atherosclerosis via Toll-like receptor signalling and NLRP3 inflammasome activation [91]. In addition, although most evidence has focused on intestinal and oral niches, other microbial ecosystems may also represent clinically relevant sources of systemic microbial exposure. The lung microbiota, altered in obesity and chronic inflammation, may contribute to circulating microbial products through increased epithelial permeability [92–95]. Similarly, skin and reproductive tract microbiota harbor diverse communities that can disseminate during barrier disruption, chronic inflammation, or invasive procedures [96]. These reservoirs should therefore be considered as complementary contributors to systemic translocation in cardiometabolic diseases.

Importantly, the concept of a viable tissue-associated microbes in internal organs remains debated, particularly because most of these tissues are low-biomass environments in which technical artefacts can easily outweigh true biological signal. A substantial part of the literature relies on 16 S/shotgun detection of bacterial DNA or RNA, which cannot by itself prove viability or sustained colonization, and may instead reflect transient microbial debris, dead bacteria, or circulating fragments captured in inflamed tissues. This point is central in low-biomass microbiomics: Salter SJ et al., demonstrated experimentally that contaminant bacterial DNA is ubiquitous in extraction kits and PCR reagents, varies between kit batches, and can dominate sequencing outputs when the true microbial load is low [97]. Extending this, Eisenhofer R et al., reviewed how both reagent contamination (“kitome”) and cross-contamination during sampling/processing lead to false-positive tissue microbiota signals, and stressed the need to interpret low-biomass findings with stringent controls and microbial-load quantification [75]. In practice, this requires parallel sequencing of extraction blanks and PCR blanks, removal of taxa enriched in controls, and causal rather than purely descriptive inference. A widely used statistical solution for this problem is provided by Davis NM, which formalizes contamination-aware pipelines (e.g., frequency- and prevalence-based filtering such as *decontam*) and shows that ignoring these steps systematically inflates “tissue microbiota” signals in low-biomass samples [98].

Moreover, even when a microbial signal is robust, distinguishing transient dissemination from true residency requires functional or longitudinal evidence. The strongest studies therefore combine orthogonal approaches that test viability, activity, and spatial localization. For viability-specific sequencing, the classic experimental foundation is Nocker A, which established that PMA selectively enters membrane-compromised (dead) cells, covalently binds DNA after light activation, and prevents PCR amplification, thereby enriching sequencing toward intact (putatively live) bacteria [99]. More recent benchmarking confirms that PMA-based methods improve viability inference in complex communities, although they still require careful optimization for tissue matrices and low loads [100]. In parallel, activity-based RNA profiling (rRNA/rRNA-derived metatranscriptomics), aseptic culture recovery, and in situ localization (FISH or related imaging) provide complementary layers of evidence. Convergent results across these modalities (load-quantified sequencing + viability assays + localization) are therefore the most convincing way to support a true viable tissue microbiota rather than a low-biomass artefact. Additional practical constraints include the persistence of procedural contamination despite rigorous aseptic workflows and the intrinsic bias of culture toward fast-growing taxa, such that culture supports viability but does not exhaustively capture tissue microbial diversity [101, 102].

Beyond these methodological limitations, an additional conceptual challenge is to distinguish transient microbial presence from true tissue residency. Bacteria or bacterial DNA may reach internal organs through low-grade bacteremia, increased vascular permeability, or passive trapping in inflamed tissues, without necessarily establishing a stable community. This difficulty has been extensively discussed in the debate on the so-called “blood microbiome”. Castillo et al. reviewed the available literature on microbial DNA in healthy human blood and concluded that many reported signals can be explained by transient bacteremia, circulating microbial fragments or technical artefacts rather than by a stable, endogenous microbiome [103]. More recently, Tan et al. analysed blood sequencing data from 9,770 healthy individuals using a contamination-aware pipeline and found no evidence for a consistent core microbial community in blood; instead, their results support a model of sporadic translocation of microbes from other body sites into the circulation [104]. However, in experimental settings, culture-based recovery of viable bacteria from freshly excised tissues—especially in the absence of any acute bacterial exposure—provides complementary evidence that goes beyond the detection of microbial DNA alone. In our study, viable bacteria were recovered from metabolic tissues after one week of diet al.one (without bacterial gavage), supporting the presence of a persistent, tissue-associated pool of viable bacteria that is amplified under metabolic stress. By analogy, we argue that claims of “live tissue microbiota” in cardiometabolic organs should be interpreted with similar caution: at present, most human data do not yet fulfil the stringent conditions required to demonstrate true residency. Therefore, while we avoid overclaiming stable “residency” in the strict ecological sense, the persistence of culturable bacteria supports more than a purely transient passage phenomenon and is consistent with chronic tissue association under metabolic stress.

Among the advanced imaging methods discussed in this review, FISH-based approaches such as CLASI-FISH have provided a major technological breakthrough for visualizing the spatial organization of complex microbial communities. Using combinatorial labelling and spectral imaging, Mark Welch et al. applied this strategy to human dental plaque and resolved, at micrometric resolution, the three-dimensional architecture of multispecies oral biofilms, revealing highly structured microbial consortia and species-specific spatial neighbourhoods within supragingival plaque [105]. While not yet demonstrated in internal organs, these approaches provide a methodological blueprint for spatially resolved validation of tissue-associated microbial signals [105].

## Experimental evidence of bacterial translocation and tissue microbiota formation

In this review, we propose that live bacteria translocate from microbiotal sites to insulin resistance–related organs such as the liver, adipose tissue and spleen. We suggest that viable bacteria can colonize these tissues and contribute to local inflammation-induced metabolic disorders. To test this hypothesis, we are conducting mouse experiments to assess the presence and viability of translocated bacteria under defined metabolic conditions. These mechanistic studies in mice serve as proof of concept before translation into humans, where further studies will be needed to confirm their clinical relevance. Building on this rationale, we performed a series of in vivo experiments designed to model both physiological and metabolic contexts of bacterial translocation. These preliminary results demonstrate that live tissue microbiota can arise spontaneously and are markedly exacerbated by metabolic stress and barrier dysfunction.

### Experimental design and induction of metabolic stress

**Fig. 1:**
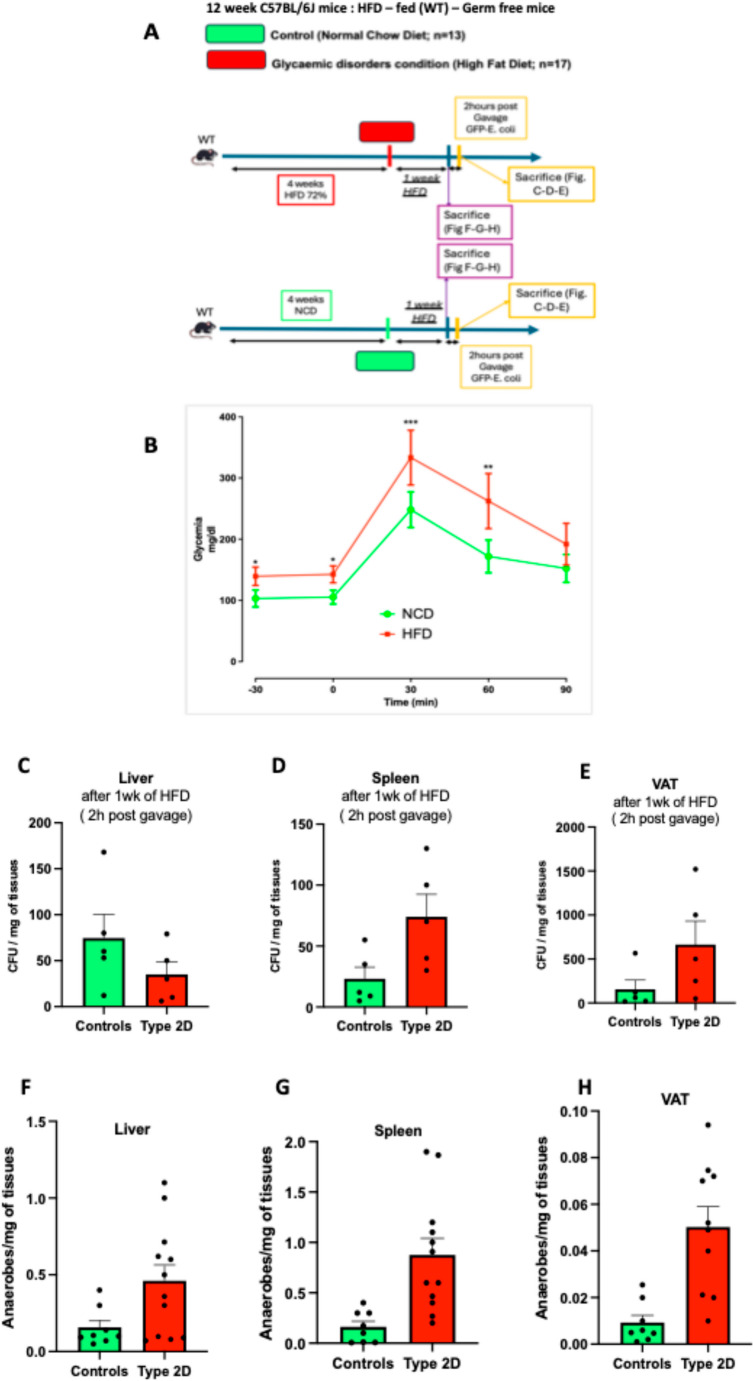

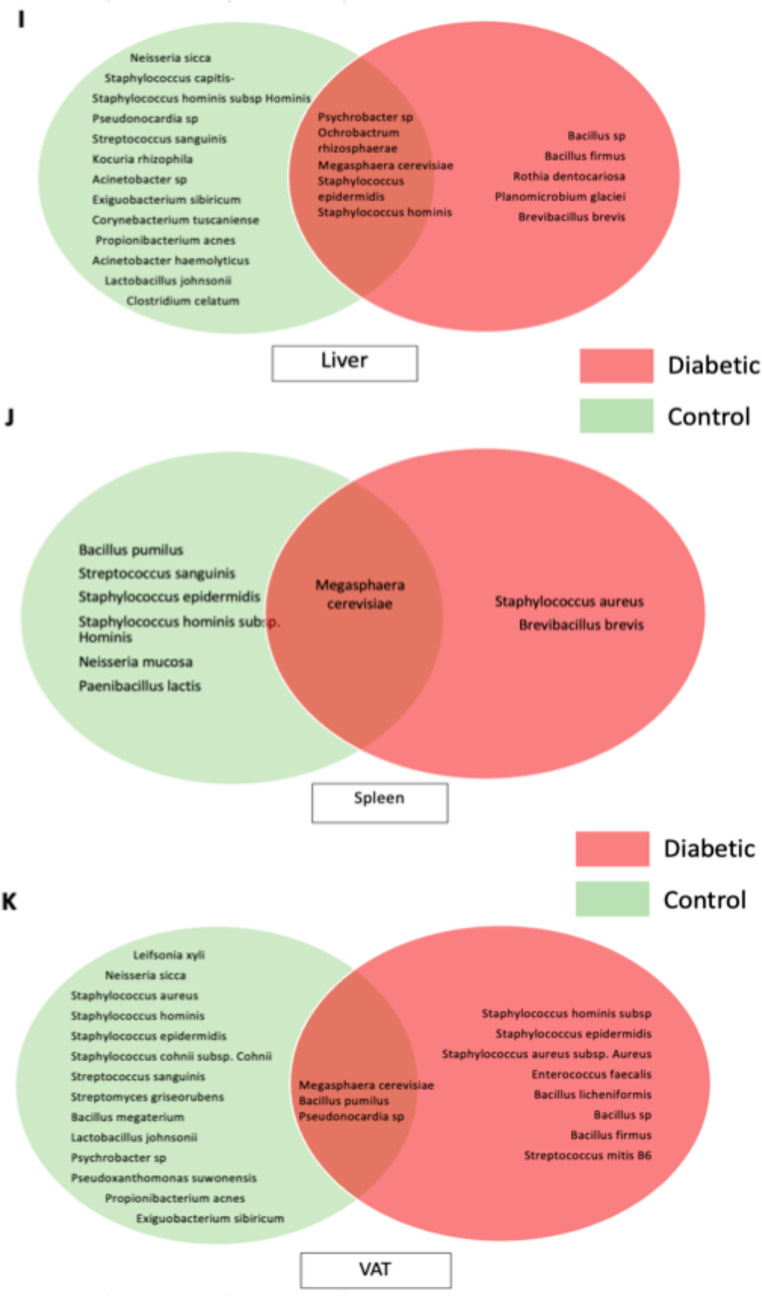
High-fat diet promotes bacterial translocation and tissue anaerobiosis in a mouse model of glycaemic dysregulation. **A** Experimental protocol performed on mice comparing the normal chow diet (NCD, *n* =13) and high-fat diet (HFD, *n* = 17) groups after 1 week of HFD **F**–**H** and after 1week HFD+2hours post gavage GFP-*E.Coli* **C**–**E**. **B** Glycaemic profiles (mg/dL) during an intraperitoneal glucose-tolerance test (IpGTT; normal chow (NCD, green bar), and high-fat diet (HFD, red bar). **C**–**E** Number of colony-forming units (CFU) in different tissues harvested from mice controls and from diabetic mice after 2h post gavaging with fluorescent *E. coli* (GFP-*E. coli*), theses data are expressed as CFU per mg of tissue (*n *= 5 in NDC/controls and *n *= 5 in HFD/type 2D). **F**–**H** CFUs counted from plates grown in anaerobic conditions onto which tissues from control and high fat diet fed diabetic mice have been placed (*n* = 8 in NDC/controls) and *n* = 12 in HFD/type 2D)). **I**–**K** venn diagrams showing cultivable bacterial taxa from liver **I**, spleen **J**, and visceral adipose tissue **K** of control (green) and diabetic (red) mice, based on 16S rRNA sequencing (*n* = 8 in NDC/controls) and *n* = 12 in HFD/type 2D). Data are shown as mean ± SEM; each dot represents one mouse

C57BL/6J mice were randomly assigned to two dietary groups: a control group receiving standard chow (NCD, *n* = 13) and an experimental group fed a high-fat diet (HFD, *n* = 17) comprising 72% fat, previously validated to induce hyperglycaemia and insulin resistance within one week (Fig. 1A). As shown in our prior studies and corroborated in Fig. 1B, short-term HFD exposure was sufficient to impair glucose tolerance, leading to elevated fasting glycemia in alignment with established literature. All animal experimental procedures were approved by the local ethical committee of the INSERM (C3155507). Data are presented as means ± SEM. Normality of data distribution was assessed using the Shapiro–Wilk test. For comparisons between two independent groups (e.g., controls vs. Type 2D, wild-type vs. ob/ob mice), an unpaired two-tailed Student’s *t*-test was applied when data followed a normal distribution. When normality was not met, the Mann–Whitney U test was used. *P* < 0.05 defined statistical significance. Statistical analyses were performed using GraphPad Prism version 10.00 (GraphPad Software, San Diego, CA).

### Assessment of acute bacterial translocation using GFP-labelled *E. coli*

To assess active bacterial translocation, we administered an ampicillin-resistant, GFP-labelled strain of *Escherichia coli* by oral gavage to both dietary groups. Two hours later, tissues including the liver, spleen and visceral adipose tissue (VAT) were aseptically harvested and homogenized. Homogenates were cultured on ampicillin-containing agar under aerobic and anaerobic conditions to identify *E.Coli* ampicillin-resistant. All recovered colonies displayed green fluorescence, confirming successful gut-to-tissue translocation of viable bacteria (*n* = 5 per group, Figs. 1C–E). Quantification of colony-forming units (CFUs) revealed a marked increase in GFP-positive CFUs in the spleen and VAT of HFD-fed mice compared to controls, while liver CFUs remained unchanged by dietary condition (*n* = 5 per group; Figs. 1C–E). These results indicate that metabolic stress selectively promotes microbial dissemination to specific tissues.

### Direct culture of tissue microbiota under basal and metabolic stress conditions

To determine whether viable bacteria persisted beyond acute exposure, we harvested tissues after one week of diet alone (without bacterial gavage). Freshly excised tissues were processed under sterile conditions and plated onto agar dishes incubated under aerobic and anaerobic conditions (*n* = 8 in NDC and *n* = 12 in HFD; Figs. 1F–H). Across all groups, viable bacterial colonies were recovered, with significantly higher CFU counts observed in the VAT of HFD-fed mice. These findings demonstrate that a chronic tissue-associated microbiota emerges during metabolic stress.

In our preliminary mouse experiments, we implemented rigorous procedures that are consistent with current best practices for low biomass microbiology. Organs were collected in an aseptic surgical field, using sterile instruments that were changed between animals and between tissues, and sampling was performed in a workspace that was physically separated from downstream microbiology procedures. Each experimental series included intra operative blanks, reagent only controls, and sterile swabs exposed to the surgical environment, in order to monitor potential sources of contamination. Tissue homogenisation and culture were carried out in a biosafety cabinet dedicated to low biomass samples, and all culture media were handled in parallel with negative controls to detect procedural artefacts. These precautions, combined with extended anaerobic incubation and the use of enriched culture media, were chosen to maximise both the reliability of sterility controls and the recovery of viable organisms when present. By explicitly applying these measures, we sought to ensure that any bacterial growth observed in our preliminary experiments reflects true biological signal rather than environmental or procedural contamination.

### Taxonomic identification and visualization of bacterial communities

To build on the observations reported in the previous figures—where culturable bacteria were detected in different tissues—we isolated representative colonies for taxonomic identification. Bacteria were extracted directly from tissue homogenates and subjected to full-length 16 S rRNA gene sequencing (1.6 kb) to precisely determine their identity. The resulting Venn diagrams illustrate distinct bacterial signatures across tissues and dietary conditions (*n* = 8 in NDC and *n* = 12 in HFD; Figs. 1I–K). The Venn diagrams provide additional insights into the qualitative differences in bacterial communities. In the liver (Fig. 1I), diabetic animals harbored unique taxa such as *Bacillus sp.*, *Rothia dentocariosa*, and *Planomicrobium glaciei*, which were absent in controls. Conversely, control livers contained species including *Neisseria sicca* and *Corynebacterium tuscaniense*. Only a limited subset of bacteria (e.g., *Staphylococcus hominis*, *Psychrobacter sp.*) was shared between groups, highlighting a selective shift in colonization patterns.

In the spleen (Fig. 1J), the diabetic group was characterized by the exclusive presence of pro-inflammatory taxa such as *Staphylococcus aureus* and *Brevibacillus brevis*, whereas controls exhibited a broader diversity including *Streptococcus sanguinis* and *Bacillus pumilus*. Only *Megasphaera cerevisiae* was common to both conditions.

The VAT (Fig. 1K) exhibited the most complex profiles. Diabetic VAT was enriched in taxa with potential pathogenicity, including *Enterococcus faecalis*, *Bacillus licheniformis*, and *Streptococcus mitis*. By contrast, control VAT displayed a greater diversity of commensal and environmental bacteria such as *Leifsonia xyli*, *Lactobacillus johnsonii*, and *Propionibacterium acnes*. The presence of shared taxa (*Megasphaera cerevisiae*, *Pseudonocardia sp.*) suggests that some bacterial populations may represent a baseline tissue-associated microbiota, while the expansion of specific groups under metabolic stress likely contributes to local inflammation and metabolic impairment.

These observations collectively support the hypothesis that metabolic conditions not only increase bacterial load but also shape the composition of tissue-resident microbiota, favouring organisms with greater inflammatory potential.

### Complementary validation in the ob/ob mouse model

**Fig. 2:**
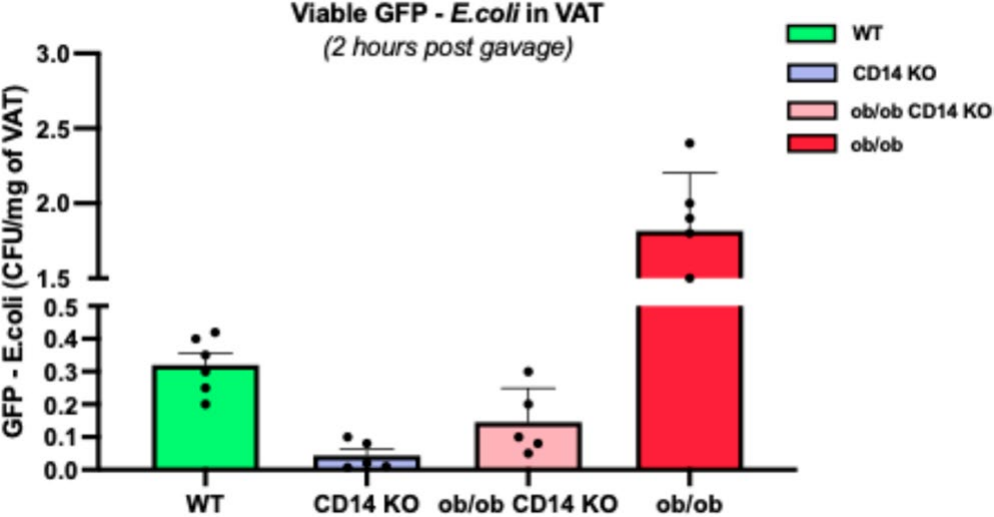
Bacterial translocation to adipose tissue in ob/ob and CD14-deficient mice. Colony-forming units (CFUs) recovered from visceral adipose tissue of wild-type control mice (*n *= 6), CD14 KO mice (*n *= 5), leptin-deficient ob/ob mice (*n *= 6), and CD14 KO ob/ob mice (*n *= 5) following oral gavage with GFP-labelled ampicillin-resistant *Escherichia coli*. Bars indicate mean CFU counts ± standard error of the mean. Samples were collected 2 hours post-gavage under sterile conditions. Data are shown as mean ± SEM; each dot represents one mouse

Strikingly, genetic ablation of CD14, encoding a critical LPS co-receptor, markedly reduced GFP–*E. coli* translocation to adipose tissue in both CD14KO (*n* = 5) and ob/ob CD14KO (*n* = 5) mice compared with their controls WT (*n* = 6) and ob/ob (*n* = 6) respectively (Fig. 2). These results implicate the LPS–CD14 signalling axis as a major driver of bacterial dissemination under metabolic stress, while the intermediate phenotype of ob/ob CD14KO mice suggests that additional CD14-independent pathways also contribute to barrier failure in severe obesity.

Together, these findings provide robust experimental evidence that:

- Bacterial translocation is dynamically regulated by diet and host genotype.
- Translocated bacteria persist beyond acute exposure and colonize metabolically active tissues.
- Specific immune pathways, notably LPS–CD14, mediate this process.

To place these experimental findings in a broader clinical context, we review key human studies that have investigated the relevance of bacterial translocation in cardiometabolic and cardiovascular diseases.

## Clinical and experimental evidence linking bacterial translocation to cardiometabolic diseases

A growing body of clinical and experimental evidence supports the notion that bacterial translocation and the establishment of live tissue microbiota contribute to the pathogenesis of cardiometabolic diseases, including obesity, type 2 diabetes, and cardiovascular disorders. This section synthesizes key studies highlighting these associations and situates our experimental findings within this evolving scientific landscape.

### Evidence from animal models

The concept of metabolic endotoxemia, first introduced by Cani et al., demonstrated that a high-fat diet induces a two- to three-fold increase in circulating lipopolysaccharide (LPS) due to gut dysbiosis and increased intestinal permeability. This low-grade endotoxemia triggers systemic inflammation, insulin resistance, and weight gain in mice, establishing a causative link between microbial products and metabolic disease [106]. Further work by Cani et al. showed that deletion of the LPS co-receptor CD14 protects mice from high-fat diet-induced metabolic disturbances, underscoring the critical role of LPS–TLR4–CD14 signalling in adipose inflammation and metabolic dysfunction [107].

Building on this foundation, Amar et al. provided compelling evidence that live commensal bacteria, including *Escherichia coli*, can translocate from the gut to mesenteric adipose tissue and the bloodstream shortly after high-fat feeding. This “metabolic bacteraemia” is dependent on pathogen-sensing pathways like NOD1 and CD14, indicating an active, regulated process rather than a passive leakage of bacterial components [21]. Subsequent interventions in mice—such as germ-free reconstitution, antibiotic treatment, and knockout models (e.g. CD14⁻/⁻, NOD1⁻/⁻)—confirm that manipulating gut microbiota, barrier integrity, or immune signalling directly influences metabolic health. These findings shift bacterial translocation from an associative observation to a functional driver of inflammation, insulin resistance, and tissue dysfunction in metabolic disease.

### Evidence from human studies in metabolic diseases

Multiple human studies have provided consistent evidence for the presence of bacterial DNA, and in some cases viable bacteria, within metabolic and cardiovascular tissues. In a pivotal study, Massier et al. analysed omental, mesenteric, and subcutaneous adipose tissues from obese and diabetic patients and detected measurable levels of bacterial DNA (ranging from 0.1 to 5 pg/µg of host DNA) [66]. Using CARD-FISH imaging, they visualized bacterial cells in adipose tissue, notably from the Proteobacteria and Firmicutes phyla. These microbial signals were strongly associated with local immune cell infiltration and elevated expression of inflammatory genes, supporting the hypothesis of a metabolically active tissue microbiota [66]. In a complementary approach, Shantaram et al. identified a diverse polymicrobial community comprising over 13 bacterial phyla in visceral adipose tissue (VAT) from obese individuals. The microbial composition was enriched in Streptococcaceae and Ruminococcaceae in obese VAT, whereas lean VAT showed higher levels of Bacilliales and Marvinbryantia. These compositional shifts were associated with neutrophil recruitment and pro-inflammatory gene expression, implicating tissue-resident microbes in VAT inflammation [108]. Further work by Rosendo-Silva et al. and Minty et al. revealed depot-specific microbial signatures that correlate with metabolic phenotypes such as insulin resistance and BMI. In addition, Minty et al. identified specific bacterial DNA signatures within adipose tissue that served as predictive biomarkers of weight loss following bariatric surgery, suggesting a potential role of the tissue microbiota in shaping postoperative metabolic outcomes [22]. These studies employed strict aseptic sampling procedures and included negative extraction controls to rule out reagent contamination, strengthening the biological validity of their findings [22, 109]. The families Porphyromonadaceae, Campylobacteraceae, Prevotellaceae, Actimomycetaceae, Veillonellaceae, Anaerivoracaceae, Fusobacteriaceae and the Clostridium family XI are signatures of the subcutaneous adipose depot while Pseudomonadaceae and Micrococcacecae characterize the visceral adipose depot. In the subcutaneous fat tissue, the abundance of Porphyromonadaceae and Family XI is positively correlated with the groups of patients with low total weight loss after bariatric surgery while the same families were correlated negatively with the high total weight loss [22].

Beyond adipose tissue, metagenomic analyses of liver biopsies from NAFLD patients have detected enriched bacterial DNA—studies consistently report taxa such as Enterobacterales, Proteobacteria, and Pseudomonadales, which correlate with disease severity and hepatic inflammation [110, 111]. In cardiovascular tissues, Koren et al. performed 16 S rRNA gene sequencing on human atherosclerotic plaques and explanted aortic valves, detecting bacterial DNA—including taxa commonly found in oral and gut microbiota such as *Streptococcus mutans* and *Enterococcus faecalis*—in patients without overt infective endocarditis, suggesting a model of chronic low-grade microbial infiltration that may contribute to vascular inflammation and plaque instability [69]. Altogether, these studies suggest that microbial translocation is not merely a post-mortem artifact or contamination event, but rather a biologically relevant process with implications for adipose, hepatic, and vascular inflammation in cardiometabolic disease.

### Evidence from human studies in cardiovascular diseases

Beyond metabolic tissues, human heart valves have also been shown to harbor bacterial DNA and even viable bacteria in the absence of clinically evident infection. In a study of explanted valves from 25 patients with structural valvular heart disease, Oberbach et al. used a combination of sonication, molecular sequencing, and microbiological culture to detect bacterial DNA in more than 50% of samples and to grow viable bacteria—including *Clostridioides difficile*, *Enterococcus faecalis*, *Staphylococcus saccharolyticus*, and *Staphylococcus haemolyticus*—in approximately 16% of cases [112]. Bacterial infiltration was confirmed histologically, suggesting that chronic colonization may contribute to valve degeneration even in non-infective settings [112]. A larger study by Di Bella et al. analyzed 34 explanted native aortic and mitral valves from patients without infective endocarditis [113]. Using next-generation 16 S rRNA sequencing with rigorous contamination controls, they detected bacterial DNA in 44% of cases. Common taxa included *Escherichia coli*, *Enterococcus spp.* (including Enterococcus faecalis), *Staphylococcus spp.*, *Pseudomonas aeruginosa*, and various oral streptococci, such as *Streptococcus mutans* [113]. Moreover, calcified valves had a higher prevalence of bacterial DNA than non-calcified ones, supporting a link between microbial presence and valve degeneration. Experimental animal models further corroborate these findings. Di Bella et al. reference earlier murine studies where oral *Streptococcus sanguis* and *Streptococcus mitis* were shown to adhere to and calcify heart valve leaflets, producing low-grade endocarditis and biomineralization without classical infection [114].

Beyond direct bacterial colonization of cardiovascular structures, recent research has also highlighted the contribution of gut microbiota-derived metabolites to systemic vascular pathology. Trimethylamine-N-oxide (TMAO), a gut microbiota-derived metabolite formed via hepatic oxidation of trimethylamine from dietary choline and carnitine, has emerged as a proatherogenic molecule. Elevated plasma TMAO levels have been repeatedly associated with increased risk of major adverse cardiovascular events—including myocardial infarction, stroke, and cardiovascular mortality. In a pivotal cross-sectional study among 227 patients undergoing cardiovascular surgery, Mafune et al. showed that individuals in the highest TMAO quartile had significantly more infarcted coronary arteries (OR 11.9, 95% CI 3.9–36.7) independent of traditional risk factors like diabetes and dyslipidemia [115]. A recent systematic review of patients post-PCI showed that elevated TMAO was significantly associated with increased risk of major adverse cardiovascular events « MACE » (HR 1.99; 95% CI 1.68–2.35) [116]. Another meta-analysis in coronary heart disease patients found a 58% increase in MACE risk (HR 1.58; 95% CI 1.35–1.84) with high TMAO levels [117]. Mechanistically, TMAO is known to affect cholesterol and bile acid metabolism, enhance foam cell formation, promote platelet hyperreactivity, impair endothelial function, and activate inflammasomes, contributing to vascular inflammation and atherogenesis [118].

In parallel, emerging evidence has highlighted that other cardiometabolic disorders, such as hypertension, are also shaped by gut microbial composition and activity. Multiple human studies have firmly linked gut microbiota dysbiosis to elevated blood pressure. For instance, Li et al. performed shotgun metagenomic profiling in a cohort of 196 Chinese adults (41 healthy, 56 prehypertensive, 99 hypertensive) and observed a Prevotella-dominated enterotype in both prehypertensive and hypertensive individuals, alongside reduced microbial diversity and distinct metabolic signatures [119]. Importantly, fecal microbiota transplantation (FMT) from hypertensive donors into germ-free mice increased recipient blood pressure, confirming a causal microbial influence on hypertension [119]. Consistent with these findings, Yan et al. reported similar microbial shifts predictive of hypertension in a matched human case-control study, including depletion of SCFA-producing taxa such as *Faecalibacterium* and *Roseburia*, and enrichment of potentially pathogenic *Klebsiella* and *Parabacteroides* [120]. A systematic review by Guo et al. further consolidated these observations, highlighting reproducible dysbiosis patterns across hypertensive human cohorts [121]. More recently, data from the large-scale HELIUS cohort showed robust associations between gut microbial taxa—particularly *Prevotella*, *Klebsiella*, and *Desulfovibrio*—and both systolic and diastolic blood pressure, independently of dietary and demographic confounders [122]. Gut-derived metabolites, particularly short-chain fatty acids (SCFAs) such as acetate, propionate, and butyrate, also play a regulatory role in human blood pressure through activation of G-protein–coupled receptors (GPR41, GPR43, GPR109A) and olfactory receptor OLFR78. These receptors modulate vascular tone, sympathetic activity, and renal sodium handling. A 2025 review by Tortelote et al. emphasized the anti-inflammatory and vasodilatory properties of SCFAs and their impact on the gut–kidney–vascular axis [123]. In a pilot clinical trial, oral sodium butyrate supplementation in untreated hypertensive patients led to modest reductions in systolic blood pressure, suggesting translational potential [124]. Mechanistic insights point toward SCFA-mediated inhibition of TH17/IL-17 A–driven inflammation and improved endothelial function, positioning the microbiota as a modifiable target in hypertension management [125].

Beyond hypertension, the role of the microbiota extends to other major cardiovascular conditions such as atherosclerosis. Atherosclerosis, the primary cause of cardiovascular disease, is characterized by lipid accumulation, chronic inflammation, and plaque formation within medium- and large-sized arteries. In a landmark study, Koren et al. performed 16 S rRNA gene sequencing on human atherosclerotic plaques and explanted aortic valves [69]. They identified bacterial DNA—including *Fusobacterium nucleatum*, *Chryseomonas*, and various oral taxa—in over one-third of samples. Importantly, the abundance of bacterial DNA correlated with macrophage-rich leukocyte infiltration, supporting a role in perpetuating local inflammation [69]. Emerging mechanistic studies have since elaborated on the contribution of *F. nucleatum* to plaque instability. In particular, Zhou et al. demonstrated that *F. nucleatum* invades the vascular intima, induces macrophage M1 polarization, enhances foam cell formation via upregulation of ABCA1 and ACAT1, and promotes plaque destabilization by elevating IL-6, IL-1β, TNF-α, MCP-1, MMP-2/8/9, and oxidized LDL [126].

Alongside bacteria, microbial metabolites also play a major role. TMAO, produced by gut flora and further processed by the liver, exacerbates atherosclerosis by promoting foam cell formation and impairing reverse cholesterol transport [127]. Elevated plasma TMAO is strongly associated with cardiovascular events and plaque burden [118]. Recent studies reinforce these findings. For instance, Chen et al. demonstrated that TMAO not only enhances foam cell formation but also promotes endothelial dysfunction through suppression of endothelial nitric oxide synthase (eNOS) and upregulation of adhesion molecules (VCAM-1, ICAM-1) [128]. Wang et al. showed that TMAO induces NLRP3 inflammasome activation, amplifying the inflammatory cascade within the plaque microenvironment [127]. Moreover, the microbial metabolite *phenylacetylglutamine* (PAGln), derived from aromatic amino acid fermentation, has been implicated in thrombosis and platelet hyperreactivity, further contributing to atherothrombotic risk [129]. Finally, microbial dysbiosis—especially oral dysbiosis with enrichment in pathogens such as *Porphyromonas gingivalis* and *F. nucleatum*—has been associated with increased arterial stiffness and systemic inflammation, supporting a gut–oral–vascular axis in atherogenesis [130].

### Integrated interpretation and translational perspectives

Collectively, data from animal and human studies delineate a continuum linking gut and oral dysbiosis, mucosal barrier disruption, bacterial translocation, and tissue colonization. Our experimental findings provide proof-of-concept that live bacteria can disseminate and persist within metabolically active tissues, and that this process is modulated by dietary and immune factors. While the detection of bacterial DNA in cardiometabolic tissues strongly supports prior microbial exposure, demonstrating true bacterial viability and metabolic activity in humans remains a challenge. Resolving this controversy will require experimental strategies that go beyond simple DNA detection. Viability oriented approaches based on RNA or labile transcripts, methods that discriminate between live and dead cells, and culture linked characterisation of isolates recovered from tissues will be essential to distinguish transient microbial remnants from truly living bacteria. In parallel, low biomass sequencing pipelines must systematically incorporate multiple negative controls, contamination aware models and quantitative assessments of microbial load, so that rare tissue associated signals can be reliably separated from background noise. Animal models colonised with defined orally or intestinally derived strains labelled with genetic barcodes or bioluminescent or fluorescent reporters could be used to track microbial translocation, persistence in cardiometabolic organs and the impact on metabolic and inflammatory phenotypes over time. For example, Fan et al. recently developed an in vivo fluorogenic labelling strategy combined with intravital two photon microscopy to visualise gut bacteria and to follow their translocation into the bloodstream and liver in obese mice, illustrating how reporter based imaging can capture the spatiotemporal dynamics of bacterial dissemination in vivo [131]. Ultimately, combining these approaches with longitudinal sampling in well phenotyped human cohorts may provide more definitive evidence as to whether a live tissue microbiota truly exists in internal organs and contributes causally to cardiometabolic disease. These insights carry important therapeutic implications. Targeting microbial translocation—by reinforcing mucosal barriers, reshaping microbial communities, or modulating host sensing pathways—represents a promising avenue to mitigate inflammation and improve metabolic outcomes. Identifying microbial signatures in tissues also offers opportunities for biomarker discovery and patient stratification.

To provide a concise overview of the clinical evidence supporting this paradigm, Table 1 summarizes representative human studies linking bacterial translocation to cardiometabolic and cardiovascular diseases. In summary, the convergence of clinical and experimental evidence supports the hypothesis that bacterial translocation and the formation of live tissue microbiota actively contribute to cardiometabolic disease pathogenesis. As this process is profoundly shaped by environmental and lifestyle factors, the following section explores these modifiable determinants and their potential for preventive and therapeutic intervention.

**Table 1:** Representative clinical studies demonstrating bacterial translocation and associated microbial signatures in cardiometabolic and cardiovascular diseases

Disease	Epidemiology	Key Species	Main Findings	Reference
Obesity	43% of adults overweight and 16% obese	*Firmicutes* (e.g., *Clostridium*), *Prevotella*, *Methanobrevibacter*	High-fat diet and dysbiotic gut microbiota promote adiposity and impair glycemic control via epigenetic mechanisms.	[22, 166, 167]
Type 2 Diabetes	520 million cases worldwide (6.1% of population, 2022)	Gram-negative bacteria	Elevated LPS and LBP in diabetic patients indicate bacterial translocation across compromised gut barrier.	[23, 92, 93, 106]
Valvular Heart Disease	50% prevalence in adults > 65 y/o; 6–13% >75 y/o	*Clostridium difficile*, *Enterococcus spp.*, *Staphylococcus spp.*, *Streptococcus spp.*, *Escherichia coli*, *Acinetobacter spp.*, *Gemella spp.*, *Lysinibacillus fusiformis*, *Mycobacterium spp.*, *Pseudomonas aeruginosa*, *Stenotrophomonas maltophilia*, *Cupriavidus spp.*	High intraindividual polymicrobial colonization in valvular disease; microbial DNA detected in 44% of valves.	[95, 151, 152]
TMAO & Coronary Artery Disease	~ 4% prevalence in France; increases to 9.5% among 65–74 y/o		Elevated serum TMAO levels associated with greater infarct burden in surgical patients.	[101, 153, 154]
Hypertension	Leading global cause of death (10 million/year); 60% prevalence > 60 y/o	*Prevotella bivia*, *Klebsiella*, *Porphyromonas*, *Actinomyces*, *Desulfovibrio*, *Enterobacter*, *Fusobacterium*, *Faecalibacterium*, *Coprobacillus*	Gut dysbiosis may influence blood pressure regulation and contribute to hypertension.	[119, 121, 168]
Atherosclerosis	Leading cause of CVD; subclinical in > 50% over 45 y/o	*Fusobacterium nucleatum*, *Chryseomonas*, oral streptococci	Oral/gut bacterial DNA found in atherosclerotic plaques; may promote macrophage activation, foam cell formation, and plaque instability.	[90, 156, 157]
Non-Alcoholic Fatty Liver Disease (NAFLD)	~ 25% global prevalence; rising in obese/diabetic individuals	*Enterobacteriaceae*, *Pseudomonadales*, *Proteobacteria*	Bacterial DNA and altered hepatic microbiota found in liver biopsies; associated with inflammation, disease severity, and endotoxemia.	[85, 158, 159]
Heart Failure	~ 26 million patients worldwide	*Streptococcus spp.*, *Enterococcus faecalis*, *Fusobacterium*, *Bacteroides*	Gut barrier dysfunction and bacterial translocation observed; elevated circulating LPS and altered SCFA metabolism associated with cardiac inflammation.	[91, 169, 170]

## Modulation of bacterial translocation by natural environmental and dietary factors: focus on vitamin D, alcohol, and gluten

The regulation of bacterial translocation across mucosal barriers is not solely dependent on the composition of the microbiota or the host’s immune status; it is also strongly influenced by environmental and nutritional factors [132, 133], such as micronutrients [134] and specific dietary components like vitamin D, alcohol, or gluten. These elements exert pleiotropic effects on epithelial integrity, immune homeostasis, and microbiota composition, thereby affecting the susceptibility to bacterial or endotoxin translocation and the development of systemic inflammation [135–138].

### Probiotics and prebiotics

Recent evidence highlights the immunomodulatory properties of specific probiotic strains, particularly those from the *Bifidobacterium* and *Lactobacillus genera*. Several *Bifidobacterium* species have been shown to promote regulatory T cell differentiation, reinforce intestinal barrier integrity, and suppress the production of pro-inflammatory cytokines, thereby supporting a balanced mucosal immune response [139]. These beneficial effects are partly mediated by interactions with epithelial and dendritic cells, modulation of Toll-like receptors (TLRs), and downregulation of the NF-κB signalling pathway. Some probiotics also enhance the production of anti-inflammatory metabolites such as butyrate or indole derivatives from tryptophan, which play key roles in maintaining gut homeostasis [140].

In parallel, prebiotics—non-digestible dietary fibers like inulin, fructo-oligosaccharides (FOS), and galacto-oligosaccharides (GOS)—selectively nourish beneficial commensal bacteria, fostering a more diverse and functional microbiota. Their fermentation by gut microbes leads to the production of short-chain fatty acids (SCFAs), including butyrate, acetate, and propionate, which exert positive effects on metabolic health, systemic inflammation, and intestinal barrier function [141, 142]. Preclinical studies have demonstrated that prebiotic supplementation improves glycaemic profiles, reduces adiposity, and lowers inflammatory markers [143, 144]. Despite promising data, both probiotic and prebiotic strategies remain investigational. Their effectiveness depends on numerous factors, including strain specificity, dosage, duration of administration, and individual host characteristics.

However, the use of probiotics is not without risks, particularly in vulnerable populations. Several clinical reports have documented adverse events such as *Lactobacillus* bacteremia and *Saccharomyces boulardii* fungemia in immunocompromised or critically ill patients, indicating that live microbes administered orally can translocate systemically under conditions of impaired barrier function [145, 146]. In a landmark trial in severe acute pancreatitis, probiotic supplementation was associated with increased mortality, underscoring that microbial interventions may have unintended deleterious effects in settings of inflammation or epithelial disruption. These examples highlight the need for careful patient selection and rigorous safety monitoring when considering probiotic-based therapies, especially in cardiometabolic conditions characterized by barrier dysfunction.

### Oral/fecal microbiota transplantation

FMT involves transferring stool from a healthy donor to a recipient with dysbiosis-related disease. While mainly used to treat recurrent *Clostridioides difficile* infections—achieving cure rates above 85%—emerging evidence suggests that FMT may also improve metabolic parameters and reduce systemic inflammation in conditions like obesity, type 2 diabetes, and cardiovascular disease. These effects are thought to result from restored microbial diversity, enhanced short-chain fatty acid (SCFA) production, and modulation of bile acid metabolism and gut barrier integrity. Some trials have reported transient improvements in insulin sensitivity and lipid metabolism following FMT from lean donors. More recently, oral microbiota transplantation (OMT)—involving transfer of oral biofilms or saliva from healthy donors—has been proposed as a complementary therapeutic to treat oral dysbiotic microbiota. Preliminary animal studies report that OMT can re-colonize the oral cavity, reduce local dysbiosis, and alter systemic microbial communities via the oral–gut axis, suggesting potential utility not only for oral health but also for metabolic and inflammatory conditions [147, 148].

Despite these promising observations, microbiota transplantation also carries important safety considerations. Several cases have documented the transmission of multidrug-resistant organisms through FMT, including extended-spectrum β-lactamase–producing *Escherichia coli* leading to severe bacteremia, prompting regulatory agencies to strengthen donor screening and material processing protocols [149]. Beyond such sentinel case reports, systematic reviews of FMT safety have catalogued both frequent but usually mild gastrointestinal adverse events and rare serious complications, including sepsis, perforation and death, underscoring that the procedure is not risk free and requires structured monitoring [150]. Adverse events such as sepsis, aspiration during delivery, and exacerbation of underlying disease have also been reported in vulnerable recipients. Even oral microbiota transplantation, although less invasive, may theoretically transmit oral pathogens associated with periodontitis or cardiovascular disease. These risks underscore the importance of standardized manufacturing, stringent donor selection, and long-term safety surveillance before widespread therapeutic implementation.

### Vitamin D: a key modulator of mucosal immunity and barrier integrity

Vitamin D plays a crucial role in maintaining mucosal barrier integrity and modulating immune responses, with deficiency increasingly linked to metabolic disorders such as type 2 diabetes, metabolic syndrome, and chronic periodontitis. Mechanistically, it enhances the expression of tight junction proteins (e.g., ZO-1, occludin) [23], boosts antimicrobial peptide production (like α-defensins), and supports regulatory T cells and ILC3 function via IL-22 pathways [151].

In preclinical models of obesity and NAFLD, vitamin D supplementation restores gut barrier integrity, reduces dysbiosis and endotoxemia, and improves metabolic and inflammatory profiles [152]. Clinically, low vitamin D levels are associated with greater periodontal inflammation and microbial imbalance. Moreover, combined vitamin D and calcium supplementation has shown anti-inflammatory and pro-apoptotic effects on adipocytes in obesity models [153, 154]. From a therapeutic standpoint, ensuring sufficient vitamin D intake represents a safe and cost-effective strategy to preserve mucosal health, potentially synergizing with dietary fibers to enhance SCFA production and promote immune tolerance.

### Alcohol: a potent disruptor of the gut–liver axis

Chronic alcohol consumption profoundly disrupts gut barrier function and immune tolerance. Ethanol metabolism generates reactive oxygen species (ROS) and acetaldehyde, which in turn impair tight junction (TJ) protein expression (e.g., claudins, occludins) and increase paracellular permeability. This “leaky gut” facilitates translocation of microbial products—including LPS—into the portal circulation, thereby activating hepatic Kupffer cells and triggering a cascade of pro-inflammatory cytokines (TNF-α, IL-6, IL-1β) that sustain systemic and liver inflammation [155]. In cirrhotic patients, bacterial translocation is markedly elevated and correlates with increased levels of lipopolysaccharide-binding protein (LBP) and IL-6, as well as clinical complications such as variceal bleeding and spontaneous bacterial peritonitis. Reiberger et al. demonstrated that non-selective beta-blockers (NSBBs) significantly reduced intestinal permeability and systemic inflammation in cirrhosis, independently of portal pressure effects. These findings suggest that targeting gut barrier function is a valid therapeutic approach in alcohol-related liver disease and broader cardiometabolic contexts [156].

Therapeutically, lifestyle interventions to reduce or eliminate alcohol consumption, combined with nutritional support and microbiota-targeted strategies (e.g., probiotics, SCFA supplementation), are likely to restore epithelial barrier integrity and mitigate systemic inflammation [157].

### Gluten: a dietary antigen and potential modulator of permeability

Although gluten is primarily implicated in celiac disease, emerging evidence suggests a broader role in modulating intestinal permeability and microbiota structure. Gluten induces the release of zonulin, a regulator of intercellular TJ disassembly, thereby increasing paracellular permeability even in individuals without overt celiac disease. Zonulin-mediated barrier dysfunction has been proposed as a contributor to bacterial translocation and systemic immune activation in various metabolic and autoimmune conditions [158, 159].

In patients with irritable bowel syndrome (IBS), a gluten-restricted diet has been shown to alleviate gastrointestinal symptoms, likely through alterations in gut microbial communities and reduced permeability. However, gluten exclusion may also decrease the intake of microbiota-accessible carbohydrates (MACs), potentially depleting beneficial taxa such as *Bifidobacterium* and *Faecalibacterium*, underscoring the need for personalized nutritional approaches [160].

From a translational perspective, the impact of gluten on bacterial translocation emphasizes the importance of balancing intestinal permeability regulation with microbial nutritional needs. The heterogeneity of patient responses to gluten withdrawal highlights the value of integrating microbiota profiling to tailor dietary interventions [161]. Recent evidence also highlights the potential of High-fiber, plant-based diets promote SCFA production (e.g., acetate, butyrate), which reinforce tight junctions and induce IL-10 and Treg responses.

Collectively, these observations underscore that modulating bacterial translocation through environmental and dietary factors—particularly vitamin D, alcohol, and gluten—represents a promising avenue for disease prevention and therapeutic innovation. These factors impact key pathways governing epithelial permeability, microbial ecology, and immune tone. In summary, the modulation of bacterial translocation through diet and environmental factors provides an accessible and promising strategy to reinforce mucosal integrity and reduce inflammation. However, these approaches should be carefully adapted to individual patient profiles to maximize efficacy and minimize unintended effects.

### TMAO reduction therapeutics

Given the deleterious effects of TMAO on cardio-metabolic health, lowering its blood levels appears to be a promising therapeutic strategy. Inhibiting TMA-lyase reduces the production of TMA (the precursor of TMAO) and thereby its subsequent conversion into TMAO. The use of 3,3-dimethyl-1-butanol (DMB), a TMAO inhibitor, has been shown—after 8 weeks of treatment in mice—to decrease circulating TMAO levels, reduce inflammation, and improve cardiac function. This represents a promising therapeutic avenue that requires further investigation through safety and efficacy studies before potential application in humans. Despite many ongoing therapeutic explorations, there is currently no validated targeted treatment for cardiovascular diseases in existing clinical guidelines, apart from lifestyle and dietary recommendations [162, 163]. However, pharmacological analyses emphasise that small molecule inhibitors of microbial TMA-lyase such as DMB remain at a preclinical stage, with no human safety data available and potential off-target effects on commensal microbial metabolism and host choline-dependent pathways, indicating that these compounds should currently be considered experimental tools rather than ready-to-use drugs in patients [164]. In parallel, recent reviews of TMAO-targeted interventions in metabolic liver disease underline that, although lowering TMAO improves experimental phenotypes in animal models, it is still uncertain whether pharmacological TMAO reduction will translate into clinical benefit in humans and whether long-term interference with this pathway might have unintended metabolic consequences [165].

## Conclusion

Over the past decade, research has increasingly challenged the classical paradigm that internal tissues are sterile under healthy conditions. Compelling experimental evidence, together with growing clinical observations, supports the concept that bacterial translocation, and the presence of tissue-associated microbial signals, including in some settings viable bacteria, may contribute to cardiometabolic disease pathogenesis. Our review highlights that dysbiosis—driven by lifestyle and dietary factors—disrupts epithelial barriers and promotes the dissemination of microbial products and, under specific conditions, viable bacteria into metabolic and cardiovascular tissues. These microbial communities can interact with local immune and metabolic pathways, fuelling chronic low-grade inflammation, insulin resistance, and tissue dysfunction. While significant progress has been made in elucidating these mechanisms, many questions remain unresolved, including the precise factors driving selective translocation, the extent to which bacteria remain viable and metabolically active in human tissues, and the causal contribution of tissue-associated microbes to disease progression under rigorous contamination-aware and multimodal validation frameworks.

Emerging strategies—including nutritional interventions, microbiota-modulating therapies (e.g., probiotics, prebiotics, oral/fecal transplantation), and targeted inhibition of microbial metabolites such as trimethylamine-N-oxide (TMAO)—represent promising avenues for prevention and treatment.

**Fig. 3:**
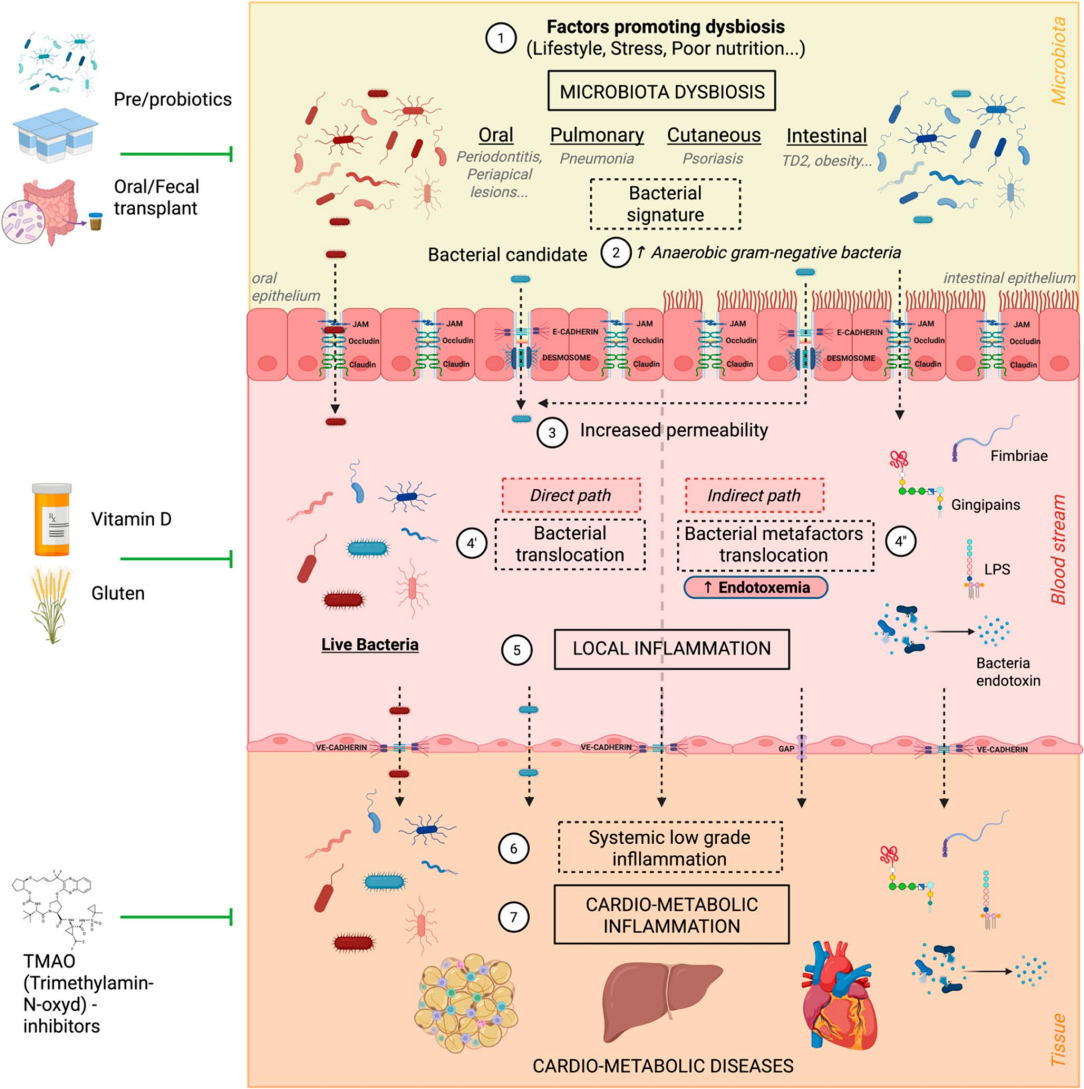
Schematic representation of the pathways linking dysbiosis, increased intestinal and oral permeability, bacterial translocation, and cardiometabolic diseases. **1** Various factors including lifestyle, stress, and poor nutrition contribute to microbiota dysbiosis across multiple mucosal and epithelial compartments, characterized by an overrepresentation of anaerobic Gram-negative bacteria. **2** Dysbiosis of the oral, pulmonary, cutaneous and intestinal microbiota, with disease-specific bacterial signatures may predispose to epithelial barrier dysfunction. **3** Dysbiosis promotes increased mucosal permeability through disruption of tight and adherens junctions (e.g., occludin, claudin, JAM, E-cadherin, VE-cadherin), facilitating the passage of microbial material. **4** Translocation of live bacteria (**4’** direct pathway) and bacterial components (**4’’** indirect pathway), such as lipopolysaccharide (LPS), fimbriae or gingipains, into the bloodstream leads to systemic exposure to microbial signals, referred to as metabolic endotoxemia. **5** Translocated bacteria and bacterial-derived products induce local endothelial inflammation, characterized by junctional disruption and increased permeability of the vascular barrier. **6** This vascular dysfunction favors the passage of microbial elements into peripheral tissues, triggering chronic systemic low-grade inflammation. **7** Persistent immune activation contributes to cardiometabolic inflammation in key target organs such as the liver and the heart, thereby promoting the development of cardiometabolic diseases. Potential therapeutic interventions targeting these mechanisms include the use of prebiotics/probiotics, oral or fecal microbiota transplantation, vitamin D supplementation, gluten exclusion, and pharmacological inhibition of TMAO (Trimethylamine-N-oxide) synthesis

To provide an integrated perspective, Fig. 3 illustrates a conceptual framework summarizing how dysbiosis, barrier dysfunction, bacterial translocation, and immune activation may converge to promote cardiometabolic diseases, and highlights potential therapeutic targets aimed at interrupting this pathogenic cascade.

## Data Availability

No datasets were generated or analysed during the current study.
